# Examining Carotenoid Metabolism Regulation and Its Role in Flower Color Variation in *Brassica rapa* L.

**DOI:** 10.3390/ijms252011164

**Published:** 2024-10-17

**Authors:** Guomei Liu, Liuyan Luo, Lin Yao, Chen Wang, Xuan Sun, Chunfang Du

**Affiliations:** 1 Agricultural College, Shanxi Agricultural University, Jinzhong 030801, China; 18388757703@163.com; 2 Cotton Research Institute, Shanxi Agricultural University, Yuncheng 044000, China; lly559919@163.com (L.L.); yaoyao302302@126.com (L.Y.); w1104chen@163.com (C.W.); yuncheng_sx2017@126.com (X.S.)

**Keywords:** *Brassica rapa* L., carotenoids, metabolome, transcriptome

## Abstract

Carotenoids are vital organic pigments that determine the color of flowers, roots, and fruits in plants, imparting them yellow, orange, and red hues. This study comprehensively analyzes carotenoid accumulation in different tissues of the *Brassica rapa* mutant “YB1”, which exhibits altered flower and root colors. Integrating physiological and biochemical assessments, transcriptome profiling, and quantitative metabolomics, we examined carotenoid accumulation in the flowers, roots, stems, and seeds of YB1 throughout its growth and development. The results indicated that carotenoids continued to accumulate in the roots and stems of YBI, especially in its cortex, throughout plant growth and development; however, the carotenoid levels in the petals decreased with progression of the flowering stage. In total, 54 carotenoid compounds were identified across tissues, with 30 being unique metabolites. Their levels correlated with the expression pattern of 22 differentially expressed genes related to carotenoid biosynthesis and degradation. Tissue-specific genes, including *CCD8* and *NCED* in flowers and *ZEP* in the roots and stems, were identified as key regulators of color variations in different plant parts. Additionally, we identified genes in the seeds that regulated the conversion of carotenoids to abscisic acid. In conclusion, this study offers valuable insights into the regulation of carotenoid metabolism in *B. rapa*, which can guide the selection and breeding of carotenoid-rich varieties.

## 1. Introduction

Carotenoids represent a class of fat-soluble, naturally occurring pigments that bestow yellow, orange, and red hues to plants. To date, over 1100 carotenoids have been identified, with a high prevalence reported in vegetables, fruits, and certain marine organisms [1,2,3]. In plants, carotenoids are present mainly in colored bodies and chloroplasts, where they perform different functions [4]. In chloroplasts, carotenoids aid in light intensification and photochemical protection, whereas in colored bodies, these pigments impart vivid colors to cells and tissues, which help in attracting insects, thereby promoting pollination and facilitating the dispersal of seeds [5]. Carotenoid metabolism in plants involves the biosynthesis and degradation pathways [6]. Specifically, the synthesis of red carotenoids and lycopene is catalyzed by enzymes such as phytoene synthase (PSY), phytoene desaturase (PDS), and carotenoid isomerase (CRTISO). Lycopene ε-cyclase (LCYE) and β-cyclase (LCYB) catalyze the synthesis of α-carotene and β-carotene, respectively; these two carotenoids are cleaved by ε-cyclohydroxylase (CHYE) and β-cyclohydroxylase (CHYB), respectively, resulting in the generation of corresponding xanthophylls. The carotenoid degradation pathway involves carotenoid cleavage dioxygenases (CCDs) or 9-cis epoxy carotenoid dioxygenases (NCEDs), which cleave carotenoids into carotenoids or abscisic acid (ABA) precursors [7]. The expression of carotenoid biosynthesis-related genes regulates coloring in the petals, rhizomes, and fruits [5,8]. PSY is a rate-limiting enzyme for carotenoid synthesis, which exerts a profound effect on coloration. As reported, inhibition of *OgPSY* transcription in Vinelandia (Oncidium “Gower Ramsey”) led to a significant decrease in carotenoid accumulation, causing a shift in the color of petals from yellow to white [1]. Conversely, *LCYE* overexpression in carrots yielded yellow coloration, while *ZDS* overexpression promoted lycopene accumulation, resulting in red fruit color. In chili peppers, mutations in β-carotene hydroxylase (CHY2) led to the accumulation of β-carotene and its derivatives, converting fruit color from red to orange [9]. Furthermore, mutations in *ZEP* reduced the activity of zeaxanthin epoxygenase, which resulted in an increase in the zeaxanthin and total carotenoid contents in chili peppers, changing their color from yellow to orange [10]

Rapeseed, belonging to Brassicaceae, is one of the most widely distributed oil crops in China and the world’s second most abundant oil crop [11,12]. Cultivated rapeseed exhibits bright yellow hues and harbors abundant bioactive compounds, including carotenoids [13,14], anthocyanins [15], flavonoids [16], and alkaloids [17], all of which are beneficial to human health. Petal color is a key agronomic trait in rapeseed, and color intensity plays a role in attracting pollinators, thereby serving as an indicator for pollination. Vivid colors attract different insect populations, facilitating pollination, pollen transmission, and species improvement, thus improving yield and quality [18,19,20]. Moreover, flower colors protect floral organs and play a role in maintaining energy balance in flower tissues under varying light conditions [21]. Among edible oils, rapeseed oil ranks third in consumption globally, lagging behind only soybean oil and palm oil [22]. Because of the abundance of bioactive compounds such as phytosterols, tocopherols, and carotenoids, rapeseed oil is considered the most beneficial and safest edible oil [22,23]. Studies have shown that rapeseed oil cultivation has a high economic value as it can mitigate soil metal pollution and improve saline-alkali land fertility [11,24]. Therefore, cultivation of rapeseed varieties with high yield, color diversity, nutritional value, and environmental adaptability holds paramount importance in agriculture.

Zhao et al. conducted transcriptome sequencing and analysis of *B. napus* with varied petal colors and observed differential expression of genes related to carotenoid content and biosynthesis pathways, revealing genes associated with carotenoid synthesis [25]. While carotenoid metabolism regulation has been studied extensively in *Arabidopsis*, maize, tomato, and other crops [26], research on the mechanism underlying its regulation in cabbage-type rapeseed plants and petal color variations in these plants remains limited. To address these gaps, in the present study, we examined the principal carotenoid constituents present in the petals, roots, and seeds of cabbage-type rapeseed plants with distinctive flower and root coloration. By integrating transcriptomic and metabolomic analyses, this study explored the regulatory mechanisms underlying variations in the carotenoid content that influences flower color in rapeseed, as well as their interplay across various tissues in these plants. The findings not only provide a theoretical foundation for uncovering the biosynthesis pathways of carotenoids in rapeseed but also serve as a reference for developing carotenoid-rich rapeseed varieties with vivid colors through molecular breeding.

## 2. Materials and Methods

### 2.1. Experimental Materials

The mutant material “YB1” and the control “TY7” were provided by the oilseed rape group of Cotton Research Institute, Shanxi Agricultural University, China. Both mutant and control plants were planted in Nanhua Farm, Yuncheng City, Shanxi Province, China. The root and stem tissues were collected for analysis before overwintering (nearly eight leaves). Petal tissues were collected at the flowering stage, and seeds were obtained at maturity. This study employed a mixed sampling method, with 3–5 plants per treatment and three biological replicates. The collected samples were immediately frozen in liquid nitrogen and stored in a −80 °C refrigerator.

### 2.2. Determination of the Total Carotenoid Content

For extraction, the samples were first ground to the powder form, and 50 mg of the powdered samples was weighed, which was then added to 0.5 mL of a hexane: acetone: ethanol mixture (1:1:1, 21 *v*/*v*/*v*). The extract was vortexed for 20 min at room temperature, followed by centrifugation at 12,000 rpm for 5 min at 4 °C. Subsequently, the supernatant was collected, and the residue was subjected to repeated extraction procedures under the same conditions. The residue was then evaporated to dryness and reconstituted in a MeOH/MTBE solution (1:1, *v*/*v*). Lastly, the solution was filtered through a 0.22 μm membrane filter for liquid chromatography–tandem mass spectrometry (LLC–MS/MS) [18,19,20].

### 2.3. Carotenoid Identification and Quantification

Carotenoids in the petals, rhizomes, and seeds of YB1 and TY7 were quantified by MetWare (Wuhan, China), and three replicates were used for each material. The extracts were analyzed using an LC-APCI-MS/MS system (UPLC, ExionLC). Carotenoid metabolites were annotated in the MetWare database and quantified through multiple reaction monitoring in each period. Mass spectrometry data were processed using Analyst 1.6.3 software [18,21].

### 2.4. Transcriptome Sequencing

The petals, rhizomes, and seeds of YB1 and TY7 were used to construct cDNA libraries. Sequencing was performed using the Illumina NEBNext^®^ UltraTM RNA Library Prep Kit platform, with three replicates per material. Raw data were filtered using fastp v. 0.19.3 and indexed using HISAT v. 2.1.0. Clean reads were compared to the reference genome (Brara_Chiifu_V3.5.fa; http://www.brassicadb.cn/#/Download/, accessed on 8 January 2022) by using DESeq2 v. 1.22.1 software to analyze differential gene expression among the three groups. *p* values were corrected using the Benjamini and Hochberg method; the corrected *p* value of ≤0.05 and |log2foldchange| ≥ 1 were set as the thresholds for significant differential expression. Kyoto Encyclopedia of Genes and Genomes (KEGG) pathway enrichment analysis was performed using GSEA-3.0.jar.

### 2.5. qRT-PCR Analysis

Total RNA was extracted from the petals, roots, and seeds of two cabbage-type rapeseed varieties by using a rapid plant total RNA extraction kit from China Shanghai Shangbao Biotechnology Co. The first cDNA strand was synthesized using a reverse transcription kit (Hifair lst Strand). For qRT-PCR analysis, 11 carotenoid synthesis-related genes in the flowers, roots, and seeds were selected; *Bra-Actin* gene was used as an internal reference (see Supplementary Table S1). The expression of genes was determined through 96-well, real-time fluorescence quantitative PCR by using the SYBR Green Master Mix kit. The reaction mixture comprised 5 μL of RNase-free water, 4 μL of cDNA, and 10 μL of Hieff qPCR SYBR Green Master Mix (No Rox), with the reaction conditions set as follows: 1 cycle at 95 °C for 3 min, and 40 cycles at 95 °C for 10 s. Gene expression levels were quantified using the 2^−ΔΔCt^ method.

## 3. Results

### 3.1. Changes in Mutant Phenotype and Total Carotenoids

**Figure 1:**
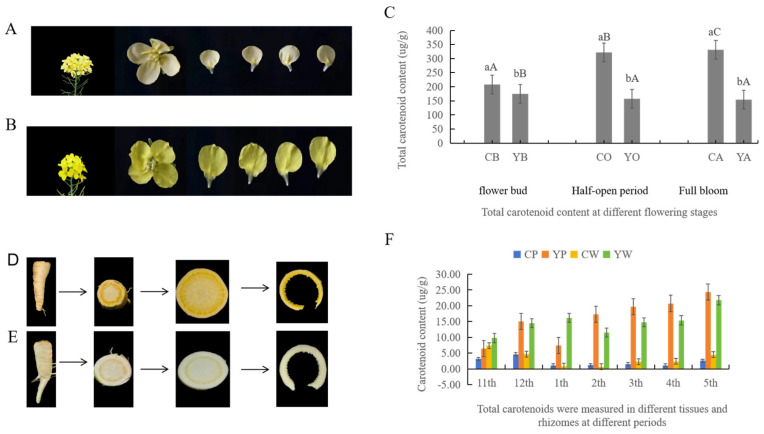
Dynamics of the phenotype and total carotenoid content in the mutants. (**A**): YB1 flowers; (**B**): TY7 flowers; (**C**): comparison of total carotenoid content at different flowering stages; CB, CO and CA represent the bud stage, semi-open stage, and full bloom stage of TY7 petals, respectively; YB, YO, and YA represent the bud stage, semi-open stage, and full bloom stage of YB1 petals, respectively; (**D**): YB1 rhizomes; (**E**): TY7 rhizomes; (**F**): comparison of total carotenoids at different stages of rhizome fertility; CP and YP denote the TY7 and YB1 cortices, respectively, and CW and YW denote the TY7 and YB1 vascular bundles, respectively; November 2022 is referred to as the 11th, December 2022 as the 12th, January 2023 as the 1st, February 2023 as the 2nd, March 2023 as the 3rd, April 2023 as the 4th, and May 2023 as the 5th. Data are expressed as the mean of three biological replicates. Differences between the two varieties were considered statistically significant at *p* < 0.05.

Phenotypic observations revealed that the mutant YB1 had a light yellow flower color (Figure 1A) and yellow rhizomes (Figure 1C), whereas the experimental group TY7 had normal yellow flower color and white root color traits (Figure 1B,D), indicating a significant phenotypic difference between the two materials. The total carotenoid content of TY7 was higher than that of YB1 during different stages of flowering. The carotenoid content of TY7 gradually increased with the progression of the flowering stage, reaching a maximum of 993.6 μg/g at the full bloom stage. However, the total carotenoid content of YB1 decreased with the progression of the flowering stage, exhibiting the value of 461.0 μg/g at the full bloom stage.

From November to the following May, total carotenoids in the rhizomes were comprehensively analyzed. Excel 2007 software was employed for data analysis, which indicated that the variations in total carotenoid contents aligned with the plant’s growth cycle. The carotenoid content in the rhizomes of YB1 exhibited a continuous increasing trend; however, the increase in the total carotenoid content in TY7 was much lower than that in YB1. This trend significantly declined in February, which may be attributed to the plant entering the winter dormancy period prior to February. Further examination of the carotenoid content in the rhizome cortex and vascular bundles revealed that the carotenoid content in the rhizomes of YB1 varied significantly between the cortex and vascular bundles during different growth stages, except for the initial growth stage of the plant and the growth transformation period in January. In TY7, carotenoid content in the cortex (in November) and the vascular bundles (in December) differed significantly compared with other growth stages of the rhizomes.

### 3.2. Quantitative Analysis of Carotenoid Metabolites

**Figure 2:**
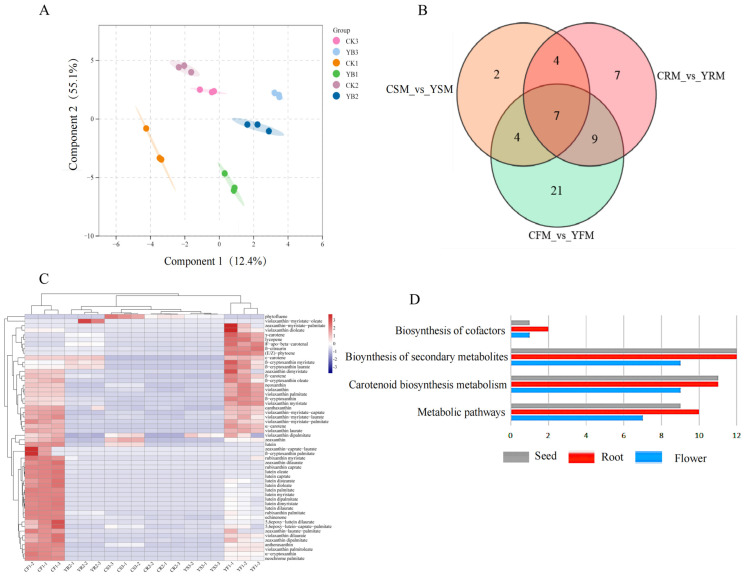
Identification and clustering analysis of carotenoid differential metabolites. (**A**): OPLS-DA supervised analysis; CK1, CK2, and CK3 denote the petal, rhizome, and seed samples of the TY7 variety, respectively; YB1, YB2, and YB3 denote the petal, rhizome, and seed samples of the YB1 variety, respectively; (**B**): metabolite Wayne plots; comparisons between CSM (TY7 seed) and YSM (YB1 seed); CRM (TY7 root) and YRM (YB1 root); and CFM (TY7 petal) and YFM (YB1 petal); (**C**): heatmap of carotenoid metabolite clustering in different tissues; CF1−1, CF1−2, and CF1−3 represent the three biological replicates of TY7 petal samples; CR2−1, CR2−2, and CR2−3 represent the three biological replicates of TY7 root samples; CS3−1, CS3−2, and CS3−3 represent the three biological replicates of TY7 seed samples; YF1−1, YF1−2, YF1−3 represent the three biological replicates of YB1 petal samples; YR2−1, YR2−2, and YR2−3 represent the three biological replicates of YB1 root samples; and YS3−1, YS3−2, and YS3−3 represent the three biological replicates of YB1 seed samples; (**D**): KEGG analysis of differential metabolites. Note: CF stands for TY7 flower, YF stands for YB1 flower, CR stands for TY7 rhizome, YR stands for YB1 rhizome, CS stands for TY7 seed, and YS denotes YB1 seed.

Utilizing targeted metabolomics, carotenoid metabolites were quantified in the petals, rhizomes, and seeds of YB1 and TY7. A total of 54 terpenoids were identified, which were categorized into two groups: carotenoids and lutein (Supplementary Table S2). The orthogonal partial least square discriminant analysis (OPLS-DA) of the samples was conducted using metabolite concentration data. The results indicated that biologically similar metabolites clustered together within a group, separated by significant distances. This indicated the reliability of the obtained metabolite data for further analysis (Figure 2A).

Subsequently, we performed Venn diagram analysis of the carotenoid metabolites detected in different tissues of YB1 and TY7. Seven metabolites, namely (E/Z)-phytoene, zeaxanthin, β-cryptoxanthin, antheraxanthin, β-citraurin, lutein myristate, and violaxanthin-myristate-caprate, were common between YB1 and TY7. Tissue-specific carotenoid metabolites, including 21 flower-specific metabolites, were identified. Of these flower-specific metabolites, 15 were downregulated and six were upregulated. In addition, seven rhizome-specific metabolites were identified, and all of them exhibited increased levels. Two seed-specific metabolites were identified and found to be downregulated (Figure 2B, Table S3).

The aforementioned 54 carotenoid metabolites were clustered and then analyzed by applying R language (R Development Core Team, New Zealand, v. R4.0). The results showed that carotenoid metabolites were abundant in flower tissues (Figure 2C), with lycopene, γ-carotene, and octahydro-lycopene being significantly more abundant in YB1; their levels were 99.1-, 56.0-, and 22.7-fold higher in YBI than in the control TY7, respectively. Conversely, TY7 exhibited higher levels of zeaxanthin, lutein, α-cryptoxanthin, antheraxanthin, lutein decanoate, lutein palmitate, and lutein myristate metabolites; the levels of these metabolites in TY7 were 2.08, 8.61, 4.33, 2.75, 18.46, 8.25, and 6.45 times higher than those in mutant YB1, respectively.

KEGG pathway enrichment analysis was performed for functional characterization of the carotenoid metabolites in different tissues (Figure 2D). The results indicated that these metabolites were significantly enriched in four pathways, namely “Biosynthesis of cofactors”, “Biosynthesis of secondary metabolites”, “Carotenoid biosynthesis metabolism”, and “Metabolic pathway”. Of the total metabolites, 9 were enriched in “Carotenoid biosynthesis metabolism” in flowers, and 11 were enriched in “Carotenoid biosynthesis metabolism” in the rhizomes and seeds (Figure 2D).

### 3.3. Transcriptome Sequencing Analysis

**Figure 3:**
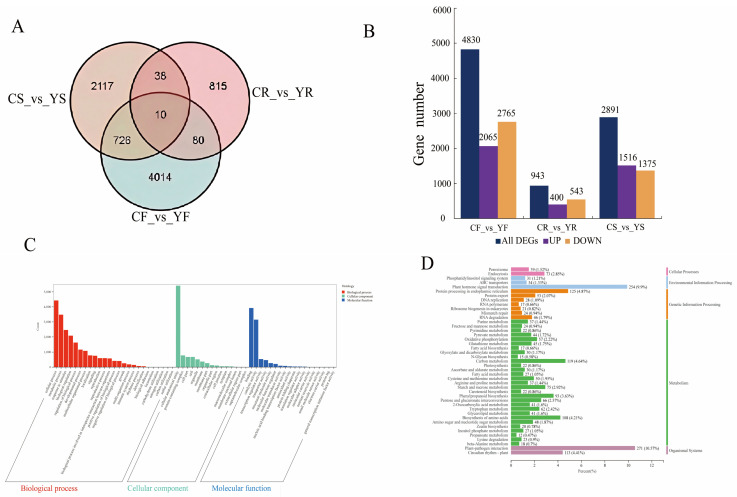
Transcriptome analysis of different samples. (**A**): Wayne plots of differentially expressed genes (DEGs) in different tissues of the control and mutant plants; (**B**): transcriptome DEGs; CF_vs._YF denotes the comparison between petals of TY7 and YB1; CR_vs._YR denotes the comparison between the roots of TY7 and YB1; and CS_vs._YS denotes the comparison between the seeds of TY7 and YB1. (**C**): GO classification of DEGs. (**D**): KEGG pathway enrichment of the DEGs.

To understand the mechanism underlying mutant flower color variations, we subjected the flowers, rhizomes, and seeds of both YB1 and TY7 to RNA-seq analysis. The sequencing results yielded clean reads exceeding 8.00 Gb in length, with the baseline percentages of Q20 and Q30 being higher than 95% and 87%, respectively, and GC content being approximately 46% (Supplementary Table S4). Venn analysis revealed a large number of differentially expressed genes (DEGs) shared between flowers and seeds; however, fewer DEGs were shared between flowers and rhizomes and between rhizomes and seeds (Figure 3A). In total, 10 DEGs were common among all three tissues. Comparative analysis revealed 42,742 genes, with the number of DEGs in the flowers, rhizomes, and seeds being 4830, 943, and 2891, respectively. In flowers, 2065 DEGs were upregulated, and 2765 were downregulated; in rhizomes, 400 DEGs were upregulated, and 543 were downregulated; and in seeds, 1516 DEGs were upregulated, and 1375 were downregulated (Figure 3B, Supplementary Table S5).

Gene ontology (GO) analysis was performed to determine the molecular and biological functions of the DEGs (Figure 3C). In the Biological Process category, the identified DEGs were enriched in 26 functions, including the cellular process and metabolic process. In the Molecular Function category, DEG enrichment was noted in 16 functions, including binding and catalytic activity. In the Cellular Component category, the DEGs were found to be enriched in 15 functions, which included mainly cellular anatomical entities and protein-containing complexes.

Further differential gene enrichment pathway analysis revealed that the DEGs in the flowers and rhizomes were significantly enriched in the biosynthesis of secondary metabolites, plant–pathogen interaction, and plant hormone signal transduction. The DEGs in the seeds were enriched mainly in the ribosomes, carbon metabolism, and oxidative phosphorylation (Figure 3D). A total of 22 DEGs were enriched in the “Carotenoid biosynthesis pathway”, and the number of DEGs varied across different tissues. Specifically, the number of DEGs in the flowers, rhizomes, and seeds was 15, 4, and 3, respectively.

### 3.4. Weighted Co-Expression Analysis of Carotenoid Metabolism-Related Genes

**Figure 4:**
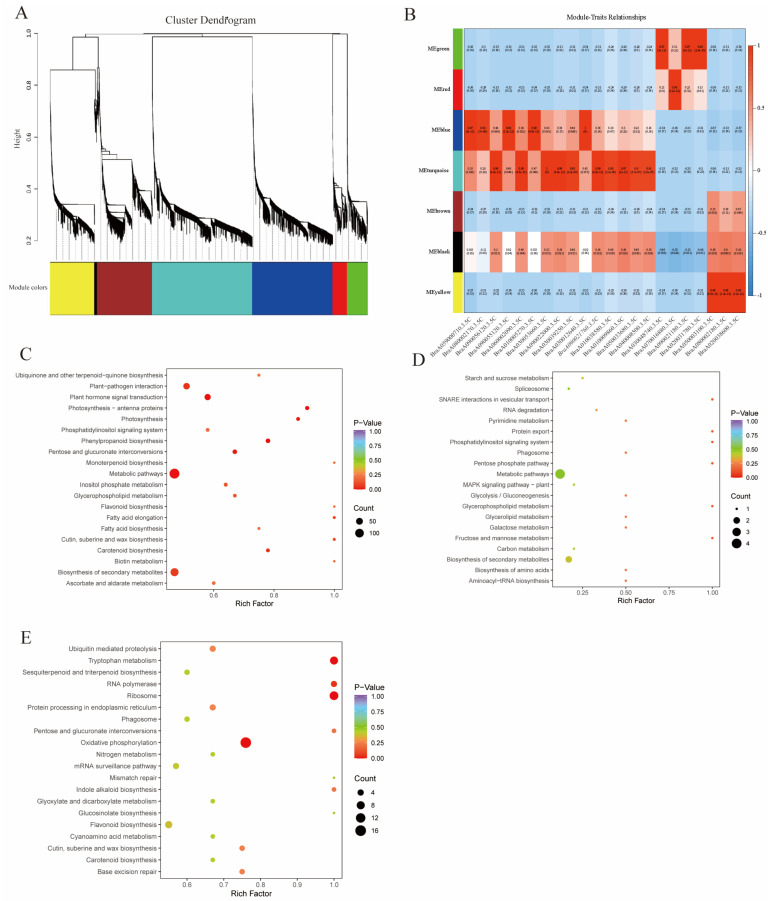
Weighted gene co-expression network analysis of the genes associated with carotenoid metabolism. (**A**): Hierarchical clustering tree diagram of co-expressed genes in WGCNA, with each leaf corresponding to one gene, and the main branches from seven modules labeled in different colors; (**B**): relationship between modules and carotenoid metabolism-related DEGs, with each row representing one module. Each column represents the carotenoid biosynthesis-related DEGs; the value of each cell at the intersection of rows and columns represents the coefficient of correlation between the modules and carotenoid metabolism DEGs (shown on the right side of the color scale), whereas the value in parentheses in each cell represents the *p* value; (**C**): KEGG enrichment analysis of turquoise module DEGs; (**D**): KEGG enrichment analysis of green module DEGs; (**E**): KEGG enrichment analysis of yellow module DEGs.

To explore the gene regulatory network for carotenoid biosynthesis in the petals, rhizomes, and seeds of the rapeseed plants, we conducted a weighted gene co-expression network analysis (WGCNA). In total, 7800 non-redundant DEGs with the fragments per kilobase of transcript per million mapped reads (FPKM) of ≥1 (Exhibit 6) were used for the analysis. These genes clustered into seven major dendrites, each representing a distinct module (labeled with different colors) (Figure 4A and Supplementary Table S7). Highly related genes clustered within the same module. Upon analyzing module–carotenoid relationships, we observed that 15 DEGs regulating carotenoid metabolism in the flowers positively correlated (correlation coefficients > 0.5 and 0.05 > *p* > 0.00) with the turquoise, blue, and black modules (Figure 4B). Additionally, four DEGs specific to the roots were found to be positively correlated with the green and red modules, and three seed-specific DEGs positively correlated with the yellow, black, and brown modules.

KEGG pathway enrichment analysis revealed that the DEGs in the turquoise and green modules were primarily enriched in the metabolic and secondary metabolite biosynthesis pathways (Figure 4C,D). In contrast, DEGs in the yellow module were mainly enriched in the oxidative phosphorylation, ribosome synthesis, and tryptophan metabolic pathways (Figure 4E).

### 3.5. Correlation Analysis between Differential Metabolites and the DEGs

**Figure 5:**
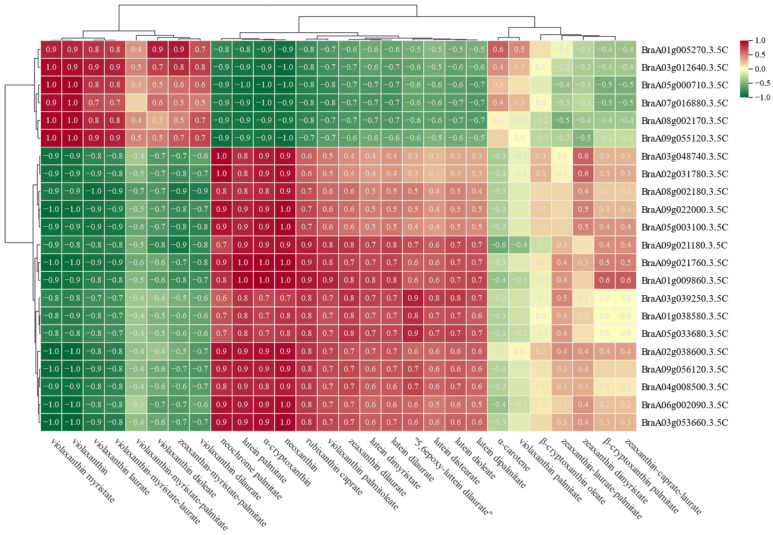
Pearson correlation analysis of DEGs with carotenoid differential metabolites (*p* < 0.05).

To gain deeper insights into the carotenoid metabolic regulatory network, we performed a Pearson correlation analysis between 30 tissue-specific metabolites and 22 carotenoid metabolism-related DEGs identified in the transcriptome analysis (Figure 5). The results showed that the purple flavin analogs (violaxanthin myristate, violaxanthin, violaxanthin laurate, violaxanthin-myristate-laurate, and violaxanthin dioleate) were associated with flower-specific DEGs (*BraA01g005270.3.5C*, *BraA03g012640.3.5C*, *BraA05g000710.3.5C*, *BraA09g055120.3.5C*, *BraA08g002170.3.5C*). Rhizome-specific DEGs including *BraA07g016880.3.5C* were significantly and positively correlated with neochrome palmitatelutein palmitate, α-cryptoxanthin, neoxanthin, and rubixanthin caprate; however, these genes were found to be significantly negatively correlated with other flavonoids including α-carotene, violaxanthin palmitate, β-cryptoxanthin oleate, and zeaxanthin-laurate-palmitate. Although these flavonoids were correlated with all DEGs, the correlations were non-significant.

### 3.6. Carotenoid Metabolism Regulatory Network for the Mutant YB1

**Figure 6:**
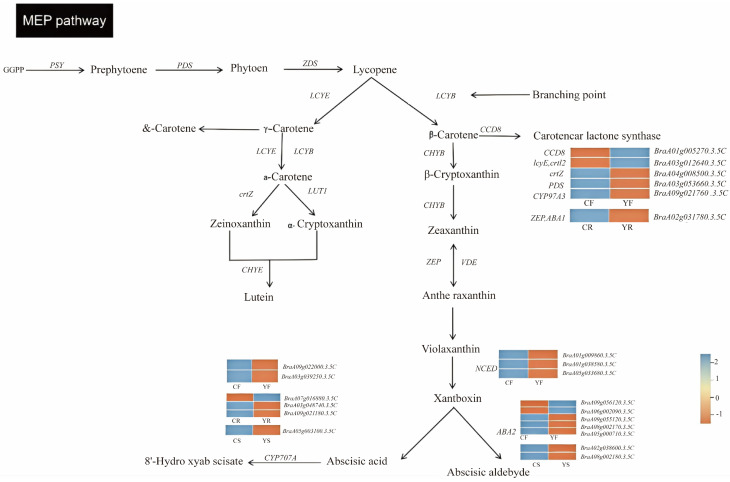
Carotenoid regulatory networks in different tissues. Note: CF: TY7 petals; YF: YB1 petals; CR: TY7 rhizomes; YR: YB1 rhizomes; CS: TY7 seeds; YS: YB1 seeds; PDS: 15-cis-octahydroxylycopene desaturase; crtL2: lycopene e-cyclase; CYP97A3: β-cyclohydroxylase; crtZ: β-carotenoids 3-lightening enzyme; CCD8: carotenoid cleavage dioxygenase; NCED: 9-cis-epoxycarotenoid dioxygenase; ABA2: xanthoxin dehydrogenase; CYP707A: (+)−abscisic acid 8′-hydroxylase; ZEP, ABA1: zeaxanthin epoxidase. Orange color indicates upregulation and light blue color indicates downregulation.

Combining the results of correlation analysis, we integrated the DEGs into the carotenoid metabolism pathway for a comprehensive analysis. In total, 22 DEGs involved in the carotenoid synthesis and degradation pathways were identified. Flower-specific DEGs were partly involved in α-carotene synthesis as well as carotenoid cleavage. Among these DEGs, one was the *CCD* gene and three were *NCED* genes; the expression of the *CCD* gene was downregulated and that of the *NCED* genes was upregulated. The expression of *ZEP*, the root-specific gene, was regulated during β-carotene synthesis, and three *CYP707A* genes in the roots were involved in ABA metabolism. All three seed-specific genes exhibited upregulation during the conversion of carotenoids to ABA (Figure 6).

### 3.7. qRT-PCR Validation

**Figure 7:**
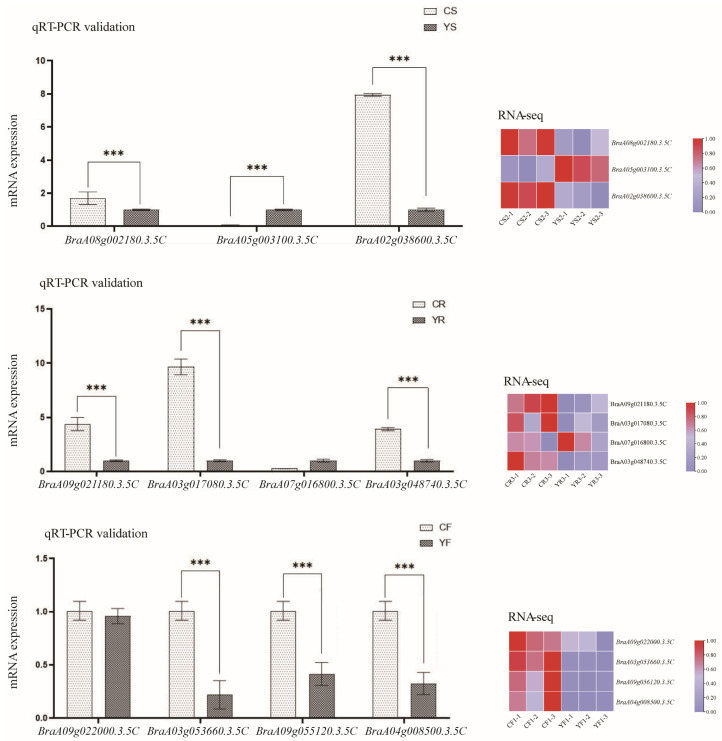
qRT-PCR assay for the differential expression profiles of genes in the seeds, petals, and roots of the control and mutant plants and transcriptome heat map. *** Significantly Note: CF: TY7 petals; YF: YB1 petals; CR: TY7 rhizomes; YR: YB1 rhizomes; CS: TY7 seeds; YS: YB1 seeds.

To confirm the accuracy of RNA-seq data, we selected 11 DEGs (Exhibit 1) for qRT-PCR assay and analyzed their expression in the petals, rhizomes, and seeds. The expression patterns of the DEGs were consistent with the transcriptome results (Figure 7).

## 4. Discussion

Carotenoids are key secondary metabolites in plants, contributing to color variations of different plant parts and playing a crucial role in plant growth and development [27]. In this study, we analyzed YB1, a cabbage-type oilseed rape mutant with light yellow flowers and yellow roots. Physiological and biochemical analyses indicated a high content of total carotenoids in YB1 rhizomes, primarily in the cortex. Quantitative analysis revealed 25 carotenoid compounds in YB1 rhizomes, significantly higher than those identified in TY7 rhizomes (12 compounds). Other carotenoid compounds, including octahydro-lycopene, ε-carotene, and lutein compounds (such as purple xanthophylls and crypto-xanthophylls), were also detected. Lutein compounds constituted nearly 75% of the total carotenoid content. Variations in lutein levels can cause changes in the color of *Chrysanthemum maniculatum* flowers from pale yellow to deep orange [28,29,30]. In this study, yellow coloration of the YB1 rhizomes may be attributed to various lutein types and their increased levels in these plants.

Physiological and biochemical analyses indicated a decreasing trend in the carotenoid content of YB1 flowers as flowering progressed. Quantitative analyses revealed abundant carotenoid compounds and their derivatives in both mutant and control plants, with YB1 having 31 compounds and TY7 having 34 compounds. Among these compounds, three were carotenoids, which exhibited increased levels in YB1, whereas the remaining compounds belonged to lutein and showed decreased levels in YB1. These findings are consistent with those of Liu et al. [31] who observed that the absence of zeaxanthin cyclo-oxygenase and excessive accumulation of lutein could induce mutation in kale-type rapeseed, imparting an orange color to the petals. KEGG pathway enrichment analysis revealed the enrichment of these compounds mainly in four pathways, namely “Biosynthesis of cofactors”, “Biosynthesis of secondary metabolites”, and “Carotenoid biosynthesis”. These findings shed light on the mechanism underlying light yellow coloration of YB1 flowers.

Plant carotenoid levels are assumed to be related to the plastid storage capacity, yet the interplay between the carotenoid synthesis and degradation rates is considered to be an indicator of total carotenoid contents in plants. Carotenoids undergo photo-oxidation or enzymatic oxidation, leading to the formation of lutein-like compounds, referred to as carotenoid cleavage products [32,33]. Enzymes catalyzing the synthesis of carotenoid cleavage products can be classified into two main categories: CCDs and NCEDs. Zhang Bao et al. examined synthetic kale-type rapeseed white-flowering lines through map cloning and reported that the white-flowering trait is governed by a single dominant nuclear gene, *BnaFC*. This gene encodes carotenoid cleavage dioxygenase (CCD4), which cleaves α- and δ-carotenes to catalyze the synthesis of α-purinone, eventually leading to flower color transition in kale-type rapeseed from yellow to white [25]. In this study, the *CCD4* gene was not differentially expressed. However, the expression of *CCD8*, a carotenoid cleavage enzyme gene, was found to be downregulated in YB1 flowers, whereas three *NCED* genes showed an increased expression. CCD8 is known to catalyze endolipid formation in solanaceous gold, affecting plant morphology by inhibiting above-ground branching [34]. In this study, the mutant YB1 exhibited a compact shape and reduced height. Thus, further investigation is needed to ascertain whether CCD8 regulates plant morphology in YBI [34]. The expression of *NCED*, which encodes a rate-limiting enzyme for abscisic acid synthesis, has been reported to be inversely related to the yellow color intensity in the petals of Hangzhou Chrysanthemum and lily, aligning with the findings of this study. Another study reported that *NCED* expression is induced under water stress, which, in turn, regulates ABA synthesis to enhance plant drought tolerance [35]. To date, no study has investigated the role of *NCED* expression in stress tolerance in rapeseed, and the results of this study may serve as a reference for future research in the field.

Despite extensive studies on the regulatory network of carotenoid metabolism, its multidimensional regulatory network remains to be completely elucidated, and this study represents an endeavor in this direction. The study revealed a series of differential metabolites and DEGs through combined transcriptome and metabolome analyses of a cabbage-type rapeseed mutant with altered floral and root color, providing a reference for further investigation into the regulatory mechanism of plant carotenoid metabolism.

## Data Availability

The datasets analyzed during the current study are available in the [NCBI] repository [http://www.ncbi.nlm.nih.gov/bioproject/1098584 accessed on 4 Month 2024] under BioProject ID: PRJNA1098584.

## Supplementary Materials

The following supporting information can be downloaded at https://www.mdpi.com/article/10.3390/ijms252011164/s1.
